# Human Amebiasis: Breaking the Paradigm?

**DOI:** 10.3390/ijerph7031105

**Published:** 2010-03-16

**Authors:** Cecilia Ximénez, Rene Cerritos, Liliana Rojas, Silvio Dolabella, Patricia Morán, Mineko Shibayama, Enrique González, Alicia Valadez, Eric Hernández, Olivia Valenzuela, Angélica Limón, Oswaldo Partida, Edwards F Silva

**Affiliations:** 1 Department of Experimental Medicine, Faculty of Medicine, National Autonomous University of Mexico (UNAM), Mexico City, 04510, Mexico; E-Mails: cerritos@miranda.ecologia.unam.mx (R.C.); rojasvelazquezliliana@gmail.com (L.R.); patricia_morans@yahoo.com (P.M.); egori1900@prodigy.net.mx (E.G.); avaladezs17@yahoo.com.mx (A.V.); ericghdz@yahoo.com.mx (E.H.); lizbthangy@yahoo.com.mx (A.L.); oswpartida@yahoo.com.mx (O.P.); 2 Department of Zoology, ENCB, National Polytechnic Institute (IPN), México City, 11340, Mexico; 3 Department of Morphology, Laboratory of Parasitology, Federal University of Sergipe, Aracaju, Sergipe, 49100-000, Brazil; E-Mail: dolabella@ufs.br; 4 Department of Infectomic and Molecular Pathogenesis, (CINVESTAV), Mexico City, 07360, Mexico; E-Mail: mineko@cinvestav.mx; 5 Department of Chemistry and Biological Science, University of Sonora (UNISON), Hermosillo, Sonora, 83000, Mexico; E-Mail: valenzuela.o@gmail.com; 6 Department of Parasitology, Institute for Biological Sciences, Federal University of Minas Gerais, Minas Gerais, Belo Horizonte, 31270-901, Brazil; E-Mail: felix@icb.ufmg.br

**Keywords:** human amebiasis, *E. histolytica*, *E. dispar*, genetic diversity, phylogeny

## Abstract

For over 30 years it has been established that the *Entamoeba histolytica* protozoan included two biologically and genetically different species, one with a pathogenic phenotype called *E. histolytica* and the other with a non-pathogenic phenotype called *Entamoeba dispar*. Both of these amoebae species can infect humans. *E. histolytica* has been considered as a potential pathogen that can cause serious damage to the large intestine (colitis, dysentery) and other extraintestinal organs, mainly the liver (amebic liver abscess), whereas *E. dispar* is a species that interacts with humans in a commensal relationship, causing no symptoms or any tissue damage. This paradigm, however, should be reconsidered or re-evaluated. In the present work, we report the detection and genotyping of *E. dispar* sequences of DNA obtained from patients with amebic liver abscesses, including the genotyping of an isolate obtained from a Brazilian patient with a clinical diagnosis of intestinal amebiasis that was previously characterized as an *E. dispar* species. The genetic diversity and phylogenetic analysis performed by our group has shown the existence of several different genotypes of *E. dispar* that can be associated to, or be potentiality responsible for intestinal or liver tissue damage, similar to that observed with *E. histolytica*.

## 1. Introduction

*Entamoeba histolytica* is the etiological agent of human amebiasis. The most common clinical forms of disease are amebic colitis and amebic liver abscess. This protozoon, once considered a unique species with different pathogenic capabilities, is the causal agent of invasive disease (pathogenic *E. histolytica*), and under circumstances that are not well defined, is able to maintain a commensal behavior in the human intestine (non-pathogenic *E. histolytica*) [1–3]. Morphological differences, such as trophozoites and cysts, between *Entamoeba dispar* and *Entamoeba histolytica* are nearly undetectable microscopically; thus, it is difficult to establish a differential diagnosis at this level. It is now clear that *E. histolytica* includes two biologically and genetically different species, one with a pathogenic phenotype called *E. histolytica* and the other with a non-pathogenic phenotype called *E. dispar* [4]. Previous to the studies that allowed the molecular characterization of both species, the estimations of the worldwide burden of amebiasis indicated that approximately 500 million people were infected by the parasite, and 10% of these individuals had invasive amebiasis. Moreover, 100,000 patients per year had died due to the clinical complications of the disease [5]. Since the separation of these two species, the epidemiology of amebiasis has changed, and we are actually just at the beginning of re-assessing the burden of this disease [6,7].

Studies on the epidemiology of amebiasis in endemic areas have utilized molecular approaches to characterize the prevalent species of *Entamoeba* in specific populations and in distinct geographic areas. These studies have characterized the diverse spectrum of human host-parasite interactions. Under this spectrum are the asymptomatic infections due to *E. histolytica* species, the asymptomatic infection due to *E. dispar*, asymptomatic mixed infections (*E. histolytica* + *E. dispar*) and symptomatic *E. histolytica* infections (intestinal or extraintestinal amebiasis). Moreover, recent studies on the genetic diversity in both the *E. histolytica* and *E. dispar* species, detected in infected individuals presenting with different outcomes of the infection (*i.e.*, asymptomatic or invasive disease), has unveiled the extraordinary polymorphism of both *Entamoeba* species [8–12]. These studies analyzed the coding and non-coding DNA regions of these species, allowing a better understanding of the heterogeneous character of the parasite infection sources as well as the complexity of the human asymptomatic infection and the disease. Recent reports showing the differences in the genotypes of *E. histolytica* isolates from fecal samples and samples from amebic liver abscesses obtained from the same patient [13] and the detection of different genotypes of *E. histolytica* detected in a patient with two simultaneous and independent amebic abscesses of the liver [14] are two examples of this complexity. Several hypotheses were considered in these studies to explain the observed genotype variations, ranging from the ingestion of inoculums containing more than one *E. histolytica* and/or *E. dispar* genotypes (strains) to the possible existence of some kind of organ tropism or a possible recombination phenomenon during intestinal colonization.

It has been reported that *E. dispar* may be the causative agent of intestinal symptoms in humans [15–17]. Using an animal model, it has also been demonstrated that this species can cause tissue lesions in the intestine and serious damage to epithelial cells [18–21]. Comparative analysis of gene content between these two species, especially those related to pathogenesis in humans, has revealed that almost the entire set of genes of *E. histolytica* is present in *E. dispar*. This includes genes such as Gal/GalNac-inhibitable lectin, the amebopore protein and different proteases [22].

In the present work, we report the detection and genotyping of *E. dispar* DNA obtained from patients with liver abscesses (amebic or pyogenic). Furthermore, we report the genotyping of an isolate obtained from a Brazilian patient clinically diagnosed with non-dysenteric amebic colitis, a strain previously characterized as an *E. dispar* species using PCR [17]. The stool specimen of the patient was cultured in Pavlova’s medium in the presence of the original intestinal flora. Finally, the phylogenetic relations of the DNA sequences obtained in our study are also discussed.

## 2. Results and Discussion

### 2.1. Patients and Analyzed Samples

Analyzed samples were obtained from 20 patients who were admitted to the department of Infectology, General Surgery and Internal Medicine at the General Hospital from the National Health Ministry in Mexico City. The presumptive diagnosis during the admission in the emergency room was hepatic abscess of unknown etiology. The final clinical diagnosis established by the primary physician was supported by the laboratory findings (thoracic X-ray, hepatic ultrasound and the presence or not of high levels of serum anti-amebic antibodies detected using ELISA [23]). In all cases, the dimensions of the abscesses required ultrasound-guided drainage. The drained material was treated for DNA extraction and PCR amplification of polymorphic regions of non-transcribed intergenic regions associated with tRNA genes using both *E. histolytica* and *E. dispar* specific primers. Six of the 20 abscess samples consistently amplified and generated a PCR product with primer Dsp1-2 (specific for *E. dispar* species), which was similar in molecular size to the PCR product obtained with the reference strain *E. dispar* SAW760; the rest of DNA samples (14) generated PCR products in the presence of *E. histolytica* specific primers (Hsp1-2) (data not shown). The general characteristics and the relevant clinical findings of these six patients are shown in Table 1. Four of the six studied samples were from female patients (age ranging from 38 to 57 years). Samples from patients with liver abscesses that were PCR positive for *E. dispar* did not show any temporal or spatial relationship, and it can be assumed that infection events were independent. The levels of serum IgG anti-amebic antibodies were tested using ELISA technique [23]. Three of the patients infected with *E. histolytica* species shown high levels of IgG anti-amebic antibodies (patients A-69, A-92 and A-104), while in the remaining three were negative (patients N-49, N-61 and N-77) (Table 1). This finding is not unexpected in individuals infected with *E. dispar* species; however, we have to remark that there is not previous experience in antibody immune response in patients with extraintestinal *E. dispar* infections. On the other hand, the ELISA test used in the present study was designed using a membrane rich extract of *E. histolytica* HM1:IMSS trophozoites, that has proved to be highly specific in detection of anti- ameba antibodies in both, serum (IgG) and saliva (IgAs) samples in patients of invasive amebiasis [23].

As we already mentioned, we also studied the ICB-ADO strain that is an isolated from a Brazilian patient of non-dysenteric amebic colitis. The stool specimen of the patient was cultured in Pavlova’s medium and maintained in a xenic culture containing the patient’s original intestinal flora. This strain was previously characterized as an *E. dispar* species using PCR [17]. In the next sections some of the phenotypic characteristics of the ICB-ADO strain are described.

The decision to perform the PCR with both sets of primers was made based on our previous experience [7] and that of Ali *et al.* (2008) [6], which suggested that the host-parasite relationship in humans due to *E. histolytica* and *E. dispar* infection may be more complex than previously thought.

### 2.2. Species Description and Phylogenetic Reconstructions

**Figure 1.**
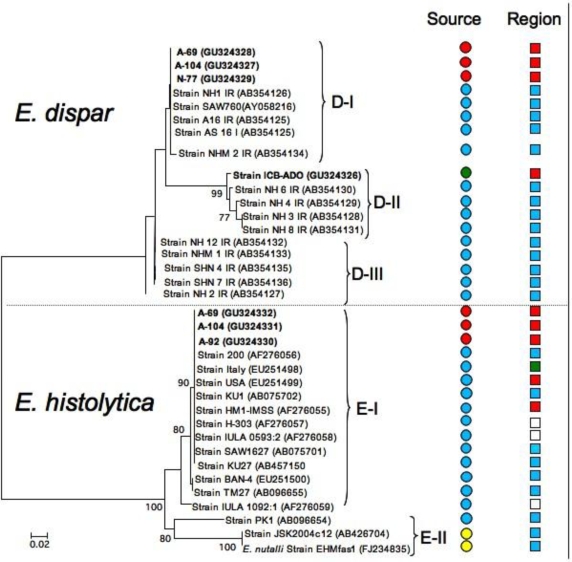
Phylogenetic reconstruction through the neighbor-joining method from intergenic tRNA Dsp1-2 sequences of *E. dispar* and Hsp1-2 of *E. histolytica.* Bootstrap values with 2500 replications are indicated close to the node numbers. Bar (0.02) shows nucleotide substitutions at each position. Colors in the first column indicate the source of samples and strain: Red, DNA of ALA; Blue, strains from fecal samples; Green, strains obtained in non-dysenteric amebic colitis; Yellow, animal source. The second column indicates the geographical region of the samples: Red, America; Blue, Asia; Green, Europe; White, data not available.

Using the molecular markers Dsp1-2 and Hsp1-2 the relationship of the samples was determined using all sequences of the intergenic region Dsp1-2 and Hsp1-2 reported in GenBank. With this data we performed a phylogenetic reconstruction of both *E. dispar* and *E. histolytica*, including sequences amplified in samples of liver abscesses. From the six sequences obtained for Dsp1-2 of liver abscess were included only three (A-69, A-104, N-77) and the remaining (A-92, N-49, N-61) was removed because they had double peaks in some positions of the sequence, which mean that more than one haplotype may be present in the sample. These double peaks can also be the consequence of the existence of a heterozygote individual or the presence of more than one individual with different genotype. The analyzed sequences, including the geographic region where the sample was obtained as well as the type of sample (*i.e.*, fecal sample, liver abscess, *etc.*), are shown in Figure 1. In the phylogenetic reconstruction of the *E. dispar* group, three main subgroups were detected: the first (subgroup D-I) includes the reference strain SAW760; the second group includes only isolates from Asian countries, particularly from Iran (subgroup D-II); the third group (subgroup D-III) includes several strains from the same geographic region. All of the analyzed sequences were obtained from stool samples. The three sequences obtained from samples of liver abscesses were included in subgroup D-I (patients A-69, A-104 and N-77), whereas the ICB-ADO strain showed a close relationship with the genealogy of subgroup D-II. With respect to the *E. histolytica* group, there were two major subgroups: the first included most of the strains of *E. histolytica* reported previously, including the reference strain HM1:IMSS (subgroup E-I), and the second subgroup (subgroup E-II) included strains isolated from animals [24] as well as the species *E. nutalli* (also of animal origin). The sequences obtained from our samples of liver abscesses were included in subgroup E-I (A-69, A-92 and A104). These sequences had a close phylogenetic relationship to the reference strain of *E. histolytica* HM1:IMSS. Although strains of *E. histolytica* have been isolated in several geographical areas, they show very little variation with respect to the *E. dispar* group.

**Figure 2.**
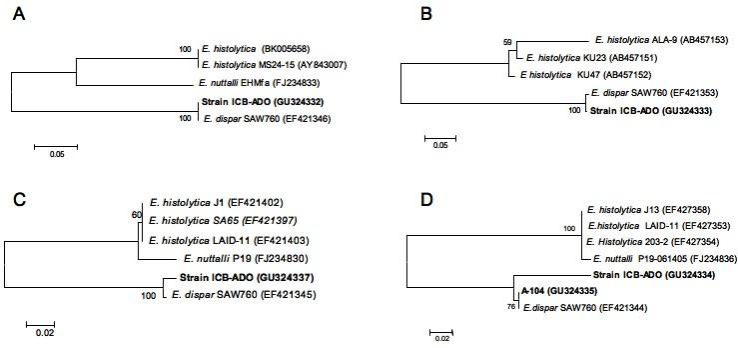
Four phylogenetic reconstructions of intergenic tRNA sequences of *E. dispar, E. histolytica* and ICB-ADO strains (A, SQD3-5; B, Dsp5-6; C, StgaD3-5; and D, NKD3-5) performed through the neighbor-joining method. Bootstraps values with 2500 replications are indicated close to the node numbers. The ICB-ADO strain is included in the *E. dispar* group in all phylogenies.

To analyze the phylogenetic relationships of the ICB-ADO strain, four different molecular markers in addition to Dsp 1-2 were amplified and sequenced to resolve its taxonomic position. The results of each of the phylogenetic reconstructions, which included sequences of *E. histolytica*, *E. dispar* and *E. nutalli*, demonstrate congruence between them. The ICB-ADO strain always appeared in the same group as the reference strain of *E. dispar* SAW760 (Figure 2). The SQD3-5 markers and Dsp5-6 (Figure 2A and 2B) showed no differences in the sequence between the ICB-ADO and *E. dispar* SAW760 strains. Based exclusively on these molecular markers, these results suggest that both strains belong to the same phylotype. With NKD3-5 and StgaD3-5 markers, however, these two strains appeared in two different outer branches (Figure 2C and 2D). Based on these reconstructions, the ICB-ADO strain could be a genetic variant of the species *E. dispar*. In addition, DNA of the liver abscesses from patient A-104, amplified with the NKD3-5 primer, generating a PCR product that, once sequenced, also clustered in the same clade of *E. dispar* species. Based on these markers, is clear that the ICB-ADO strain and A-104 sample are variants of the species *E. dispar*.

### 2.3. Genetic Diversity

The genetic diversity of *E. dispar* and *E. histolytica* analyzed using the Dsp1-2 and Hsp1-2 molecular marker (250 bp) showed a high genetic diversity in the *E. dispar* group. This was in contrast with the diversity observed in the *E. histolytica* group. The numbers of haplotypes detected in samples of ALA from the *E. dispar* group (Table 2) showed high diversity compared to the ALA of the *E. histolytic* group.

Although we have to mention that in the case of *E. histolytica* and *E. dispar* species, there is not an antecedent of the genetic diversity measured by the segregating sites and the rates of π and θ, values of genetic diversity observed in the two groups constructed with *E. dispar* sequences can be considered high, in accordance with previous reports where the number of haplotypes was estimated [10,11,25] (Table 2). In the group that included only liver abscess samples analyzed in this study, using four sequences we obtained three haplotypes, 33 segregating sites and values of π and θ of 0.065 and 0.071, respectively. When the analysis was performed with all the sequences registered in GenBank of the *E. dispar* group, we obtained 15 different haplotypes from 19 sequences, with 53 total segregating sites (Table 2). In the two groups with *E. histolytica*, we found that for the group that included only sequences of amebic liver abscesses, the genetic diversity was very low, showing only two haplotypes of three different sequences and only one segregating site. The diversity of the total sample of *E. histolytica* available in GenBank data base was very different from that found in *E. dispar*; in this case, we only detected 7 haplotypes of 17 sequences with 49 segregating sites and a very low π value with respect to θ. The relationships between these two indexes indicate that the diversity in this group is due to only a few sequences that have nearly all the segregating sites. Comparing the genetic diversity of these two species in the group of patients with liver abscesses, it can be observed that *E. dispar* displays a high genetic diversity in relation to group of *E. histolytica* (Table 2).

A number of studies have demonstrated the high genetic diversity of *E. dispar* and *E. histolyica* as well as the evolutionary dynamics of these two species [10,26]. In a study conducted by Mojarad *et al.* [27], it was shown that the number of different genotypes of *E. dispar* isolated from asymptomatic cyst passers was very high. In 28 isolates 12 new genotypes were found, a finding consistent with in *E. dipar* isolates from asymptomatic cyst passers in Mexico [12]. In the present work, the *E. dispar* detected for the first time in cases of liver abscesses were also found to be highly polymorphic.

### 2.4. Hypothetical Considerations to Explain the Presence of E. dispar in Amebic Abscess of the Liver

**Figure 3.**
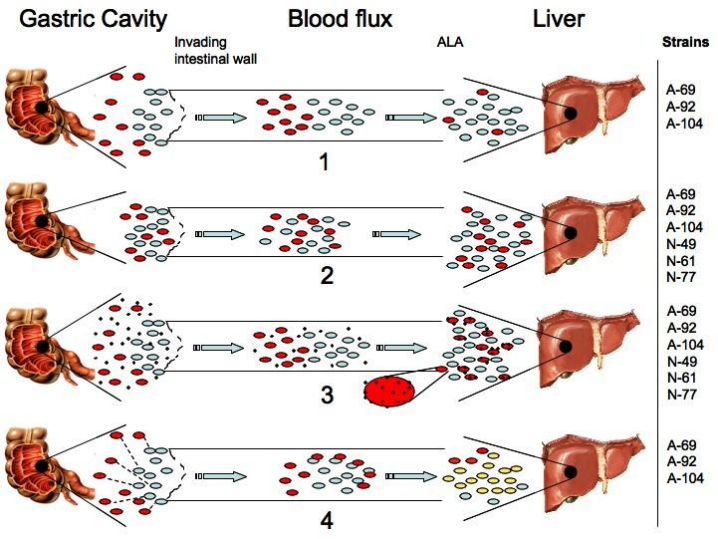
Four different hypotheses may explain the presence of *E. dispar* in liver abscesses. First, *E. histolytica* (blue) species invade the intestinal mucosa, producing erosion and ulceration of intestinal tissue. Both species are then placed in portal circulation and seeded into the liver. *E. dispar* (red) does not produce tissue damage but may take advantage of the pathogenic capacity of the *E. histolytica* species (1). The second possible explanation proposes that the two species are similarly responsible of both the intestinal and liver damage (2). The third hypothesis deals with bacteria-mediated pathogenicity. This suggests that the pathogenesis of at least some *E. dispar* strains may be mediated by (Black points) a type of bacterial flora in a particular host (3). The fourth hypothesis (Table 2) suggest that recombination events between *E. histolytica* and *E. dispar* species. In the right column are included the samples that could represent the possible hypothetical scenarios.

The presence of DNA sequences of *E. dispar* in drainage samples of amebic liver abscesses in humans leads us to consider four different hypothetical scenarios. The first is based on a primary condition, that is, the necessary intestinal co-infection of the patient by a highly virulent *E. histolytica* and *E. dispar* species. Both species colonize the intestinal mucosa, and the invasive strain of the *E. histolytica* species displays all the known pathogenic strategies to induce the tissue damage of the intestinal epithelium. The *E. histolytica* trophozoites produce the ulceration of deeper intestinal tissue that may allow trophozoites to reach the blood capillary network and then migrate to the portal circulatory system. Both *E. histolytica* and *E. dispar* trophozoites could then be seeded into the hepatic parenchyma. This scenario considers that *E. dispar* is moving by portal circulation into the liver with no active involvement in intestinal tissue damage. Once *E. dispar* trophozoites are in the hepatic parenchyma they take advantage of the environment created by *E. histolytica* trophozoites. It is possible that the initial density of the *E. dispar* population in the liver may be very low in relation to that of *E. histolytica*, and *E. dispar* survival in this environment may depend on the damage presumably caused by *E. histolytica* (Figure 3A).

The second hypothesis proposes that in the human host, under particular circumstances, *E. dispar* and *E. histolytica* are indeed pathogenic in both the colonic mucosa and the liver parenchyma. The dynamics of the infection indicate that the two species produce epithelial damage in the intestinal lumen and that both can migrate to and invade the liver, which presents with the known tissue damage done by both parasites. Under this hypothesis, the population densities of both species (in the intestine or the liver) may depend on their particular degree of virulence. In a study conducted by Costa *et al.* [17], it was mention that two isolates of the species *E. dispar* obtained from Brazilian patients can induce amebic liver abscesses in experimental animals [17,27]. Their results showed that both of the tested isolates (ICB-ADO and MGL) can produce similar cytopathic effects and amebic liver abscesses compared to those produced by a highly virulent *E. histolytica* EGG strain [17].

On the other hand, *in vitro* and *in vivo* experimental models have suggested the potential pathogenic behavior of *E. dispar*, indicating that this species can produce damage to the intestinal or hepatic organs. Nevertheless, both species can display pathogenic and non-pathogenic behaviors under particular circumstances. The outcome of the host-parasite relationship in the human amebiasis may therefore result in an asymptomatic infection (asymptomatic cysts passers) or an invasive disease (intestinal amebiasis, amebic liver abscess) [18–21].

The third hypothetical scenario proposes that *E. dispar* could maintain symbiotic associations with bacteria from the intestinal flora, considering that the type of intestinal flora may enable or enhance the infectivity of *E. dispar* in either the intestinal lumen or the liver. Recent studies have demonstrated that isolates of *E. dispar* and *E. histolytica* in xenic or monoxenic culture conditions can increase their virulence, causing larger liver abscesses in laboratory animals in comparison to *E. histolytica* maintained in axenic culture conditions [17,27].

In endemic areas of amebiasis, the co-infection with other intestinal pathogens (bacteria or parasites) is more a rule rather than an exception. With this in mind, it is highly possible that interactions of *Entamoeba* species with both bacteria of intestinal flora [17,27–29] and/or pathogenic bacteria, may modify the infectious behavior of both (*i.e.*, the parasite and the enterobacteria). Some pathogenic enterobacteria have genes that encode for molecules associated directly (pathogenicity island) [30,31] or indirectly (induction of inflammatory responses) [32,33] with tissue damage in the intestine or extraintestinal organs, such as the liver. Under these circumstances, it is likely that *E. dispar* and *E. histolytica*, both excellent phagocytes organisms, may ingest bacteria or attach bacteria to their membrane surface and allow the expression of *Entamoeba* or bacteria virulence molecules. This would lead to simultaneous tissue damage or functional alterations. The possible molecular mechanisms to explain this circumstance can be the lateral transference of pathogenic enterobacteria genes to *Entamoeba* species, or the induction of expression or overexpression of both bacteria and/or *Entamoeba* pathogenic genes. We have to mention that bacterial lateral gene transfer particularly in *E. histolytica* species, has been previously reported [34–36]. On the other hand, the induction or increase in virulence of *E. histolytica* HM1:IMSS mediated by the presence of pathogenic enterobacteria has been recently described in full detail by Galvan-Moroyoki *et al.* (2008) [37]. They showed that the effect of *Shigella dysenteriae* and *Escherichia coli* (EPEC) increased the cytopathic effect on epithelial cells, cysteine proteinase activity and the expression of Gal/GalNac lectin on the *E. histolytica* membrane surface and that these effects are enhanced when *E. histolytica* phagocytosed bacteria. One of their relevant findings was the interplay between bacteria-epithelial cells, which made the epithelial cells more susceptible to damage from *E. histolytica.* This in particular induced the expression of the pro-inflammatory interleukin IL-8, which is chemiotactic for neutrophils and *E. histolytica* trophozoites. In contrast, under the same conditions, the *E. dispar* SAW760 strain was not affected and did not display any pathogenic behavior. The authors suggest that the infection with pathogen enterobacteria may prepare the intestinal epithelial cells, making them susceptible to the mechanism of virulence displayed by *E. histolytica*. The evidence of the pathogenicity of the *E. dispar* ICB-ADO strain isolated from a patient with a clinical diagnosis of non-dysenteric amebic colitis, and tested in *in vitro* studies and through the induction of amebic liver abscesses in the hamster model, displays a variety of the pathogenic effects observed in the *E. histolytica* infection. As we already mentioned, the *E. dispar* ICB-ADO strain used in our experiments was maintained in a xenic culture containing the original intestinal flora. This culture was previously tested for the induction of amebic liver abscesses, including the simultaneous induction of the abscess with virulent axenic trofozoites of *E. histolytica* HM1:IMSS as a positive control. In contrast, the induction of liver abscesses with the bacterial flora and without trophozoites did not produce hepatic damage [17].

We must describe the differences between our results and those reported by Galvan-Moroyoqui *et al.* [37], particularly in relation to the failure to induce pathogenic behavior in the *E. dispar* SAW760 mediated by *S. dysenteriae* and *Escherichia coli* (EPEC). As mentioned, the ICB-ADO strain was isolated from a patient of non-dysenteric amebic colitis maintained in a xenic culture containing the patient’s original intestinal flora. In contrast, *E. dispar* SAW760 was isolated from an asymptomatic cyst passer, and the strain was maintained under axenic conditions. Furthermore, the cytopathic studies carried out with the ICB-ADO strain showed a positive cytopathic effect on epithelial cell monolayers and on the induction of amebic liver abscesses in hamsters. In addition, the assays utilizing another Brazilian isolate from an asymptomatic patient (MGL) maintained under xenic conditions also induced amebic liver abscesses. This strain was characterized as *E. dispar* zymodem 1 [17,27]. Moreover, in the presence of *Crithidia fasciculate*, both the ICB-ADO and MGL strains failed to produce amebic liver abscesses under monoxenic conditions in the hamster model [17,27]. The evidence described above support the participation of intestinal bacteria, pathogenic or not, in the induction or modulation of the virulent phenotype of the *Entamoeba* species. Are these conditions fundamentally important in the expression of a pathogenic phenotype in the *Entamoeba* species in humans? This is a question to be addressed in the near future. Nevertheless, it should be noted that clinically, three of the six patients were diagnosed with pyogenic liver abscesses and one as a mixed liver abscess. Only two patients were clinically diagnosed with amebic liver abscesses. However, the characteristic of the drained material correlated with the purulent material used for clinical diagnosis; unfortunately, the bacteriological study and it results were unavailable. Even though, these data are particularly suggestive of a participation of bacteria in tissue damage, we have to acknowledge that, our results are related with DNA sequences of intergenic regions associated to tRNA of *Entamoeba* species, not bacterial genes, which do not prove or disapprove the proposed scenario.

The fourth hypothesis is indeed supported by results of the estimation of genetic diversity (Table 2), suggesting that *E. histolytica* infection may be a clonal infection while *E. dispar* is considerably diverse, which point out to possible recombination events between *Entamoeba* species. If we consider that the genetic limits between both *E. histolytica* and *E. dispar* species are tenuous and the hybridization phenomena possible, we should be able to find some of pathogenic factors of *E. histolytica* in the genetic background of *E. dispar*, this is not a minor subject, if we take into account that several of the pathogenic genes detected in *E. histolytica* are also present in *E. dispar*, at least in the reference strains *E. histolytica* HM1:IMSS and *E. dispar* SAW760, besides, these strains are only two more strains in the vast and diverse *Entamoeba* species population.

## 3. Experimental Section

### 3.1. DNA Extraction and Molecular Markers (Targets)

With the purpose of cleaning the sample and removing mucus from the liver abscess drainage, 200–500 μL of the sample were placed in 500 μL of MgSO_4_ 0.1 M. Thereafter, 50 μL l of proteinase K was added and incubated at 56 ºC for 30 min. DNA was then extracted using the “DNA Easy Tissue” Kit (Qiagen, Valencia, CA, USA) following the manufacturer’s instructions. DNA from the ICB-ADO strain was obtained from 10^6^ trophozoites harvested from a xenic culture using the TRIZOL reagent kit (Invitrogen, Life Technology, Carlsbad, CA, USA) following the manufacturer’s instructions.

Five different tRNA gene-linked short tandem repeats were amplified in both DNA from liver abscess drainage and from trophozoites of the ICB-ADO strain using primers species-specific for *E. dispar* and *E. histolytica* as previously described [38]. Dsp1-2 and Hsp1-2 locus from different liver abscesses were amplified using Dsp1 and Dsp2 for *E. dispar* and Hsp1 and Hsp2 for *E. histolytica*. The reaction and conditions for Dsp1-2 and Hsp1-2 loci was identical. A total volume of 20 μL reaction was prepared with 1 U of Taq polymerase (Roche: Diagnostics Gmbh Mannheim, Germany), 5.0 mM of MgCl_2_, 1 mM of dNTP, 2 μM of each primer and 2 μL of DNA 25–100 ng/μL. The PCR conditions were as follows: 5 min at 95 °C for the initial incubation, followed by 30 cycles of 30 sec at 95 °C, 30 s at 55 °C and 30 s at 72 °C and a final extension step of 10 min at 72 °C. To obtain a greater quantity of PCR product, we made a re-amplification under the same conditions using 1 μL of the amplified product. Dsp1-2 and Hsp1-2 PCR products size are 430 pb and 340 pb respectively. The PCR re-amplification product was purified using the “Gel Extraction DNA kit (Qiagen Valencia, CA, USA). The selected bands (the same size of the *E. dispar* SAW760 and *E. histolytica* HM1:IMSS) were cut and sequenced with the same primers used in PCR amplification.

The other molecular markers were used to amplify only DNA of the ICB-ADO strain using both species-specific primers for *E. dispar* (Dsp5-6, SQD3-5, StgaD3-5 and NKD3-5) and *E. histolytica* (Hsp5-6, SQH3-5, StgaH3-5 and NKH3-5). The reaction and PCR conditions for amplification of these molecular targets was previously described [38].

### 3.2. Phylogenetic Reconstruction for Different Molecular Markers

The sequencing reactions had a total volume of 15 μL consisting of 2 μL of the Big Dye Terminator Sequencing kit (Applied Biosystems), 1.6 μM of primer and 5 μL of the purified amplified product. The amplification conditions were: 1 cycle of 5 min at 95 °C, 45 cycles of 10 s at 95 °C, 10 s at 50 °C and 4 min at 60 °C. Sequencing was done in a capillary sequencer (ABI-Avant 100).

Sequences were manually verified with the BioEdit program [39]. Taxonomic identity was established by comparing the obtained sequences against the GenBank (NCBI) data. Sequences were aligned using the ClustalX software program [40]. Phylogenetic reconstruction for molecular markers Dsp1-2 and Hsp1-2, Dsp5-6, SQ, Stga and NK was carried out through the neighbor-joining method using the MEGA program, version 3.0 [41]. The substitution model for each one of the markers was Kimura 2-P.

### 3.3. Population Genetics Analysis

Through the use of sequences of Dsp1-2 and Hsp1-2, different parameters of population genetics were analyzed. We made four different groups for diversity genetics analysis: sequences of *E. dispar* obtained from liver abscesses analyzed in the present study, all sequences of the species *E. dispar* available in GenBank data base, sequences of *E. histolytica* obtained from liver abscesses in this study and the total sample of sequences from *E. histolytica* accessed in GenBank. The number of segregant sites, the number of haplotypes per group, the mean nucleotide diversity per site (π) and the expected variation per site under the neutral evolution assumption (θ) were obtained using DnaSP version 5.0 [42].

### 3.4. Nucleotide Sequence Accession Numbers

The partial sequences of the different samples of liver abscesses and the ICB-ADO strain determined in this study have been deposited in GenBank under accession numbers GU324326 to GU324337.

## 4. Conclusions

The present results undoubtedly demonstrate the existence of different genotypes of the *E. dispar* species that can reach the liver during an invasive amebiasis event. Whether this *Entamoeba* species is playing an active role in the hepatic tissue damage observed in amebic liver abscesses or if it is only a passive participant in the described damage needs to be determined systematically. The evidence presented here is not simply anecdotic, as our data indicate; the phenomenon is real and more frequent than previously assumed. In our opinion, this is not a circumstance restricted to Mexico. It is likely that this phenomenon can also be observed in other endemic areas.

The proposed hypothetical scenarios are not merely speculative and can be reasonable approached; some studies have demonstrated that the third hypotheses may explain the presence of *E. dispar* in liver abscesses of patients studied in this work [27]. On the other hand, by utilizing molecular sequencing techniques and using five different molecular markers, it was shown that the ICB-ADO strain, which is responsible for the intestinal damage in the colon of the studied patient and later tested in laboratory animals, is a genotype of the species *E. dispar.* Hypothesis 2 and 4 are indeed supported by the present results (Table 2). These scenarios are not contradictory and can be almost simultaneously approached.

Finally, the high genetic diversity found in both samples of genotypes from liver abscesses as the total sample of *E. dispar* (Table 2) suggests that recombination, genetic differentiation and/or differential selection processes may be operating in this species. Further analysis to estimate the frequency of this event in other patients with liver abscesses is required to determine the dynamics of infection and co-infection by *Entamoeba* and the interaction with bacterial flora and/or bacterial pathogens in endemic areas of amebiasis. As mentioned, it should be noted that co-infections due to intestinal parasites and gastrointestinal bacterial pathogens are the rule and not the exception. It is clear that the key approach to a rational control of this health problem is multidisciplinary epidemiological research. Are we at the beginning of a change in the paradigm regarding to the pathogenicity of both *Entamoeba* species? In this sense, there is sufficient evidence to consider *E. dispar* as a potential agent capable of inducing tissue damage in the digestive tract and liver in humans.

## Figures and Tables

**Table 1.** Clinical data of patients with liver abscesses that were PCR-positive for molecular markers Hsp1-2. and/or Dsp1-2 It is noted that abscesses clinically diagnosed as pyogenic were PCR positive for *E. dispar* species.

Sample Code	CD^1^	Gender	Age	ELISA^2^	PCR-MD^3^
A-69	MLA	F	57	0.63	*E.h, E.d*
A-92	ALA	F	39	1.1	*E.h, E.d*
A-104	ALA	M	40	1.1	*E.h, E.d*
N-49	PLA	F	52	0.14	*E.d*
N-61	PLA	F	38	0.47	*E.d*
N-77	PLA	M	55	0.27	*E.d*

1 Clinical diagnosis: MLA, mixed liver abscess (amebic and pyogenic); ALA, amebic liver abscess; PLA, pyogenic liver abscess.

2 Levels of IgG anti-amebic antibodies, values represent the O.D. at 490 nm. Cut off value 0.52.

3 Molecular diagnosis: *E.h, Entamoeba histolytica; E.d*, *Entamoeba dispar*.

**Table 2.** Parameters related to the genetic diversity of four different groups of the species *E. dispar* and *E. histolytica* using Dsp1-2 and Hsp1-2 molecular markers (250 bp). In contrast to the *E. histolytica* group, there was high genetic diversity in the *E. dispar* group. The number of haplotypes detected in samples of amebic liver abscess (ALA) of the *E. dispar* group showed a high diversity compared to the ALA of the *E. histolytic* group.

Group^1^	No. Sequences	No. Haplotypes	Ss^2^	π	θ
*E. dispar* ALA	4	3	33	0.065	0.071
*E. dispar* total	19	15	53	0.081	0.064
*E. histolytica* ALA	3	2	1	0.002	0.002
*E. histolytica* total	17	7	49	0.034	0.069

1 *E. dispar* ALA and *E. histolytica* ALA: Number of samples of liver abscesses obtained in this study; *E. dispar* total and *E. histolytica* total: number of analyzed sequences available in the GeneBank data base

2 Number of segregated sites.
